# Cable-free brain imaging for multiple free-moving animals with miniature wireless microscopes

**DOI:** 10.1117/1.JBO.28.2.026503

**Published:** 2023-02-10

**Authors:** Yangzhen Wang, Zhongtian Ma, Wenzhao Li, Feng Su, Chong Wang, Wei Xiong, Changhui Li, Chen Zhang

**Affiliations:** a Capital Medical University, School of Basic Medical Sciences, Advanced Innovation Center for Human Brain Protection, Beijing Key Laboratory of Neural Regeneration and Repair, Department of Neurobiology, Beijing, China; b Tsinghua University, School of Life Sciences, Beijing, China; c Peking University, College of Future Technology, Department of Biomedical Engineering, Beijing, China; d Peking University, Academy for Advanced Interdisciplinary Studies, Peking-Tsinghua Center for Life Sciences, Beijing, China; e Beihang University, School of Biological Science and Medical Engineering, Beijing, China; f Peking University, National Biomedical Imaging Center, Beijing, China; g Chinese Institute for Brain Research, Beijing, China

**Keywords:** microscope, neuroimaging, free-moving

## Abstract

**Significance:**

Although several miniature microscope systems have been developed to allow researchers to image brain neuron activities of free moving rodents, they generally require a long cable connecting to the miniature microscope. It not only limits the behavior of the animal, but also makes it challenging to study multiple animals simultaneously.

**Aim:**

The aim of this work is to develop a fully wireless miniature microscope that would break constraints from the connecting cables so that the animals could move completely freely, allowing neuroscience researchers to study more of animals’ behaviors simultaneously, such as social behavior.

**Approach:**

We present a wireless mini-microscope (wScope) that enables simultaneously real-time brain imaging preview from multiple free-moving animals. The wScope has a mass of 2.7 g and a maximum frame rate of 25 Hz at 750  μm×450  μm field of view with 1.8-μm resolution.

**Results:**

The performance of the wScope is validated via real-time imaging of the cerebral blood flow and the activity of neurons in the primary visual cortex (V1) of different mice.

**Conclusions:**

The wScope provides a powerful tool for brain imaging of multiple free moving animals in their much larger spaces and more naturalistic environments.

## 1 Introduction

Recording the activity of neurons in freely behaving rodents is a crucial way for neuroscientists to understand how neuronal networks process information in naturally and ethologically relevant behavior. Many electrophysiological techniques have been presented to record a large number of neurons, such as silicon probes and multi-tetrode arrays.^1^ However, it is challenging to differentiate each neuron within the sampled population by the shapes of waveform. In 2011, the Ghosh et al. developed the first wide-field miniature microscope (mini-scope) for measuring neuronal activity; the device had a mass of 1.9 g, a lateral resolution of 2.5  μm, and a frame rate of 30 fps at 640×480  pixels.^1^ Later, Cai et al. initiated the UCLA Miniscope project, featuring an open-source imaging platform with a mass of <3  g and a frame rate of 30 fps at 752×480  pixels.^2^ With the help of the UCLA platform, researchers have developed a variety of mini-scopes. For example, Skocek et al. used a microlens array and a constrained matrix factorization strategy to image a volume of $700\times 600\times 360\;{\mu m}^{3}$ at a frame rate of 16 Hz and an axial resolution of 30  μm with a device weighing >4  g.^3^ Using a transparent polymer skull implant and a light-emitting diode (LED) array, the Rynes et al. developed a whole-cortex mini-scope with a field of view (FOV) of 8×10  mm, a mass of 3.8 g, and spatial resolution ranging from 39 to 56  μm.^4^ These and other mini-scopes have provided researchers with powerful tools to study the neural circuits of many sub regions of the brain, such as the cortex, hypothalamus, hippocampus, striatum and amygdala.^5^^–^^9^ However, the tethered cables used in these mini-scopes are easily to be tangled, which makes it challenge to image multiple free-moving mice simultaneously or in a covered area (such as caves). Therefore, to study the behaviors of multiple mice in a single arena with more naturalistic conditions simultaneously, it would be ideal to develop a total wireless mini-scope system without tethered cables.

To meet this demand, several wireless mini-scope systems have been reported to record the activity of neurons in rodents.^10^^,^^11^ Unlike aforementioned mini-scopes with tethered cables, the images from these microscopes are temporally stored on a Micro SD card attached to the miniscope. While these systems enable mice to roam untethered during experiments, systems with SD card have relatively higher weight (3.8 to 4.5 g), lower resolution (≤320×320  pixels), and lower frame rate (≤20  fps). The increased weight of these microscopes could influence the behavior of the animal, and the imaging qualities are not comparable with that of wired mini-scopes. In addition, the important real-time preview capability is lost and those frames stored in SD microscopes card can only be viewed offline after the experiment. Although another wireless mini-scope without SD card was developed,^5^ no rodent experiment has been reported yet using that system. Therefore, it is desired to have a lightweight wireless mini-scope with high performance and real-time preview function to image the brain of freely moving mice.

Here, we developed a lightweight, wireless mini-scope, named wScope, for brain imaging in freely behaving mice. There are a lot of technical challenges to keep the light weight while maintain performance level comparable to that of a wired microscope, including low power, heating dissipation, environmental interference, etc. Over much iteration, the current wScope used frequency modulation (FM) technology to transmit high-quality image signals wirelessly. Besides real-time previewing capability, our system enables wireless control of imaging parameters, including camera gain and LED power. Our system supports simultaneous brain imaging of up to 8 mice in the same arena. A lightweight, 100-mAh lithium battery provides sufficient power for 15-min continuous recording. In addition, to save power to record more valuable data, our wScope also has a unique function to switch between standby and active modes through wireless remote control, which substantially extends the lifetime of the experiment by consuming energy only when needed. Table 1 shows the comparison of our wScope with other reported wireless miniscopes, in which the first two scopes (including ours) do not use SD cards, while the last two scopes use SD.

**Table 1:** A comparison with other wireless mini-scope systems.

	wScope (this work)	Liberti et al.^5^	Barbera et al.^11^	Shuman et al.^10^
Body weight (g)^a^	2.7	1.8^b^	3.9	4.5
Resolution	640 × 480	640 × 480	200 × 200	320 × 320
Frame rate (Hz)	25	30	10	20
FOV	700 μm×450 μm	800 μm×600 μm	500 μm×500 μm	700 μm×450 μm
Multiple-animal study	Yes	Not reported	Yes	Not reported
Live viewing	Yes	Yes	No	No
Remote control	Yes	No	No	No
Rodent experiment	Yes	No	Yes	Yes

a All body weights do not contain the battery weight.

b This value does not count in the weight of the wireless transmission module.

We evaluated the imaging and signal transmission performance of wScope and recorded the cerebral blood flow (CBF) and neuronal activity of the primary visual cortex (V1) in different freely moving mice, both in an open space and in a tunnel, the latter of which is impossible with a tethered cable. The kinetics of vessel diameter change and neuronal calcium transients were recorded reliably by wScope, denoted a high performance of wScope for brain imaging. Our results demonstrate the wScope system as a powerful tool to study CBF and neural circuits in multiple naturally behaving mice. The wScopes can help research topics such as group social behavior and cerebral disease in complex, custom-designed environments.

## 2 Methods and Materials

### 2.1 Design of the Wireless Mini-Microscope System

**Fig. 1:**
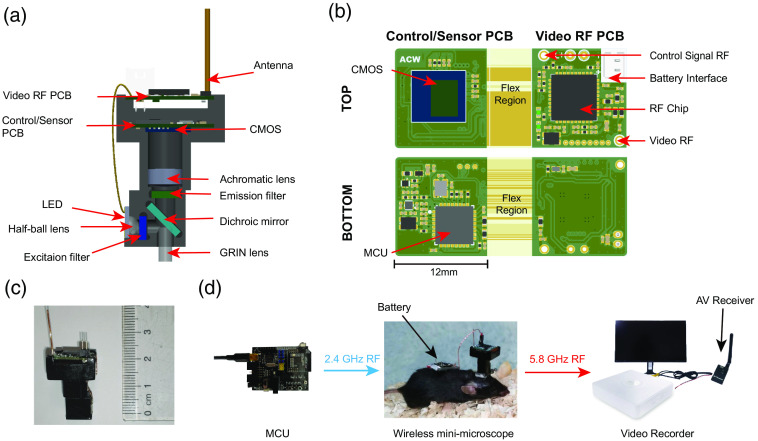
Wireless mini-scope imaging system. (a) Design of the wScope. (b) Custom-built video RF and control/sensor PCBs, with views of the top and bottom components. (c) Image of an assembled wireless microscope. (d) Overview of the wireless microscopy system.

The overall shell and optics of our system are based on the open-source, mini-microscope system developed at UCLA,^2^ with our specially designed new electrical circuit part. The entire microscope has a volume of $∼2.8\;{\mathrm{cm}}^{3}$ and a mass of 2.7 g, and it is powered by a lithium battery mounted on the back of the mouse. The internal and circuit design of the microscope is shown in Figs. 1(a) and 1(b).

The developed wScope has a blue LED with a spectral peak at ∼475  nm (LXML-PB01-0040). The excitation light from the LED is first collimated by a half-ball lens before passing through an excitation filter (Chroma, ET470/40x, $3.5\times 4\times 1\;{\mathrm{mm}}^{3}$), and then reflected into the objective GRIN lens by a dichroic mirror (Chroma, T495lpxr, $4\times 6\times 1\;{\mathrm{mm}}^{3}$). The emitted fluorescence light collected by the objective GRIN lens sequentially passes through the dichroic mirror and the emission filter (Chroma, ET525/50m, $4\times 4\times 1\;{\mathrm{mm}}^{3}$), and finally forms the image onto a CMOS sensor (OV7960, Omni Vision Technologies, Inc) via an achromatic lens (Edmund Optics, 5-mm diameter, 15-mm FL).

As shown in Fig. 1(b), the customized printed circuit board (PCB) contains two parts connected by a flexible connector, which is folded to form spatially separated two layers, upper layer and lower layer. The upper circuit board includes a radio frequency (RF) chip; an antenna contacts for receiving control signals and transmitting video signals; and interfaces for power LED. The main components of the lower circuit board are a CMOS image sensor and a 2.4-GHz RF transceiver with an embedded microcontroller unit. This folded two-layer design not only reduces the amount of space needed but also effectively facilitates heat dissipation. No significant behavioral difference was observed in mice whether they wore the wScope or not.

The OV7960 image sensor generates streaming pixel data at a constant frame rate of 25 Hz with analog output. The image frames are wirelessly transmitted using an RTC6705, a 5.8-GHz band FM transmitter, which are received by an analog video receiver and then viewed and saved in real time using a video recorder [Fig. 1(d)].

Our system uses nRF24LE1 chips (Nordic Semiconductor), a 2.4-GHz RF transceiver with embedded microcontroller, to realize wireless system control. Several key parameters, including the CMOS gain, the brightness of the excitation LED, and the system standby function, can be controlled remotely. Each wScope is assigned a unique ID number so that its parameters can be controlled separately during the experiment without affecting other wScopes. The controller is a customized board that has another nRF24LE1 installed, which receives commands from the computer through a serial port (Fig. S1 in the Supplementary Material). The development board sends the instructions wirelessly using a 2.4-GHz RF signal.

Besides wireless image transmission and system control, our circuit board also reserved the wired connection ports to make the mini-scope can work as a wired version. This function helps system test and performance comparison.

### 2.2 System Performance Testing

**Fig. 2:**
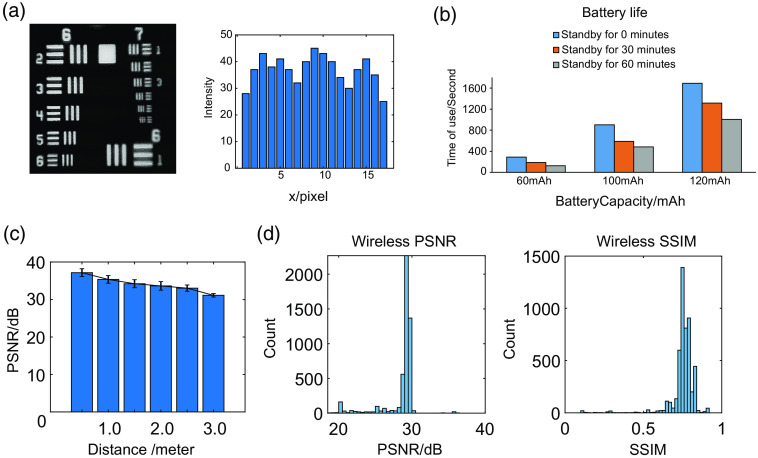
Characterization test of the system. (a) Imaging of a USAF1951 Resolution Test Target by the wScope. 2.19  μm can be resolved experimentally from the cross-sectional profile along the red line in left. (b) Battery continuous working times after different standby time. (c) PSNR at different distances. (d) Histogram of per frame PSNR and SSIM of the wireless conditions, as compared to the wired condition (at 2 m).

The imaging resolution is measured by imaging a USAF1951 Resolution Test Target, as shown in Fig. 2(a), which demonstrates a ∼2-μm resolution.

We measured continuous working time of batteries with different capacities (Table S1 in the Supplementary Material). Additionally, to verify the effectiveness of the standby function in conservation of battery life, we also measured the working time after half an hour and one hour of standby, respectively [Fig. 2(b)]. Balanced with battery life and weight, we chose a lithium battery with a capacity of 100 mAh, a mass of 1.7 g, and a size of $4\;\mathrm{mm}\times 10\times 25\;{\mathrm{mm}}^{3}$ for the *in vivo* experiment.

To evaluate the wireless image transmission quality of our wScope, we employed two parameters, namely, the peak signal-to-noise ratio (PSNR) and structural similarity (SSIM), to quantify the similarity between images by wireless and wired mini-scopes.^12^^,^^13^ To calculate the PSNR, we first calculate the mean squared error (MSE). For two m × n images, x (the wired image) and y (the wireless image), the MSE is defined as

$$\mathrm{MSE}=\frac{1}{mn}\overset{m-1}{\underset{i=0}{\sum }}\overset{n-1}{\underset{j=0}{\sum }}{\left[x(i,j)-y(i,j)\right]}^{2}.$$ (1)

The PSNR is calculated from the MSE with the following formula:

$$\mathrm{PSNR}=10*\log(\frac{{\mathrm{MAX}}^{2}}{\mathrm{MSE}})=20*\log(\frac{\mathrm{MAX}}{\sqrt{\mathrm{MSE}}}).$$ (2)

MAX is the maximum value of the color scale that is applied to each point in the image. If each sampling point is 8 bits, then MAX is 255. The smaller the MSE is, the larger the PSNR, and the better the image quality. Then, the SSIM between x and y can be calculated as follows:

$$\mathrm{SSIM}(x,y)=\frac{\left(2\mu_{x}\mu_{y}+c_{1})(2\sigma_{xy}+c_{2}\right)}{\left(\mu_{x}^{2}+\mu_{y}^{2}+c_{1})(\sigma_{x}^{2}+\sigma_{y}^{2}+c_{2}\right)},$$ (3)

where $\mu_{x}$ is the average of image x, and $\mu_{y}$ is the average of image y. $\sigma_{x}^{2}$ is the variance of x, $\sigma_{y}^{2}$ is the variance of y, and $\sigma_{xy}^{2}$ is the covariance of x and y. $c_{1}={\left(k_{1}L\right)}^{2}$, $c_{2}={\left(k_{2}L\right)}^{2}$, $k_{1}=0.01$, and $k_{2}=0.03$.^14^ The larger the SSIM value, the more similar the two images are; when the two images are exactly the same, the SSIM is equal to 1.

We evaluated the image quality as a function of the distance between wScope and the wireless receiver, as shown in Fig. 2(c). Taking the image at 0 distance as the reference image, we took images at 0.5-m intervals from 0 to 3 m, and the calculated PSNR values were all above 30. Thus, our system can guarantee image quality at a distance of 3 m.

To test the quality of the wireless transmission, we use the wScope to image the fluorescence micrometer and sent and analyzed video signals via both wired and wireless transmission to evaluate the quality of the images obtained in real time. Our wireless miniscope reserved the port for wired connection to be function like a traditional wired version, which is very useful for system debug and test. We also experimentally confirmed that the wired connect won’t affect simultaneously wireless image transmission. Therefore, when we compare the imaging quality between wireless and wired modes, instead of doing one after another, we connected the cable with the miniscope and acquire images from wired and wireless modes simultaneously. The PSNR value of the image received after wireless transmission was ∼28 to 30 dB, and the SSIM value was ∼0.7 to 0.8 [Fig. 2(d)], which demonstrates that our wireless transmission can guarantee reliable image quality.^12^^,^^15^^,^^16^

## 3 Experimental Results

### 3.1 Animal Surgery

All animal experiments were conducted at Capital Medical University Laboratory Animal Center, an Association for Assessment and Accreditation of Laboratory Animal Care-approved animal facility. All animal experiments were undertaken in accordance with the Guide for the Care and Use of Laboratory Animals (eighth edition), and all experimental protocols were approved by the Institutional Animal Care and Use Committee of Capital Medical University. Wild-type C57BL/6 mice (male, aged 8–12 weeks) were purchased from Vital River Laboratories (Beijing, China) and maintained on a 12/12-h reversed dark-light cycle. All experiments were performed during the light cycle. Before surgery, the mice were anesthetized with tribromoethanol (240  mg/kg, Sigma), and a craniotomy was performed over the primary visual cortex of the right hemisphere (coordinates with respect to bregma: anteroposterior, −2.8  mm; mediolateral, 2.5 mm; and dorsoventral, 0.2 mm). After the craniotomy, a 1.8-mm diameter GRIN lens (Edmund Optics) was placed on the surface of the cortex without damaging the tissue. The gap between the GRIN lens and the skull was covered with Kwik-Sil (World Precision Instruments) to protect the cortex. A metal baseplate was mounted on the skull using cyanoacrylate adhesive and dental acrylic. After surgery, the animals were allowed to recover for 1 week, during which they were intraperitoneally injected with ceftriaxone sodium (200  mg/kg) and dexamethasone (5  mg/kg) every day to prevent inflammation and edema. One week after the surgery, the mice mounted with a 3D printed dummy microscope on their heads, for rousting the locomotor behavior.^17^ After training, the mice showed robust locomotor behavior (Fig. S2 in the Supplementary Material).

### 3.2 Experimental Area

**Fig. 3:**
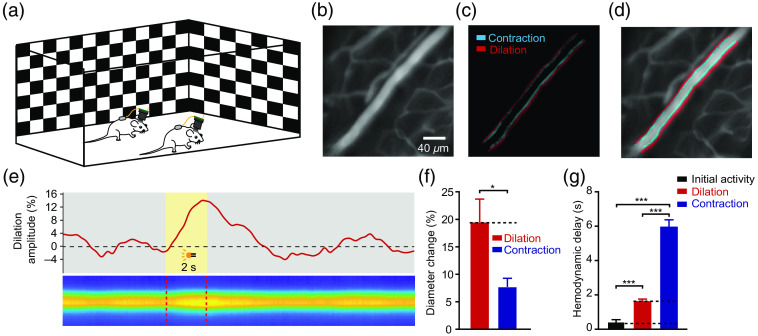
*In vivo* CBF imaging in freely moving mice. (a) Illustration of mice fitted with the wScope system in an open arena. (b) Example image of brain vessel imaging using the wireless microscope system. (c) Changes in the diameter of the vessel during visual stimulation. (d) Merged image showing the vessel and the diameter change. (e) Example trace (c1) and line scan heatmap (c2) of vessel diameter change during stimulus presentation. (f) The absolute diameter changes for vessel dilation and contraction during visual stimulation. (g) The delays of the initial phase, the peak of the dilation phase and the peak of the contraction phase of vessels with respect to the onset of visual stimulation.

To evaluate the performance of the wScope system for in vivo brain imaging, we built an arena and placed a camera above it to monitor the behavior of the animals. The size of this experimental area is 50×50  cm. Two sides of the arena were made of transparent acrylic plates, allowing the animals to see visual stimuli presented on screens outside the transparent walls [Fig. 3(a)].

### 3.3 Cerebral Vessel Imaging in Awake Mice

Before imaging, the animals were anesthetized with isoflurane. Fluorescein isothiocyanate-dextran (2% w/v, MW 70,000, 50 ml, Sigma) was administered into the caudal vein to label the blood plasma. A wScope was mounted on the mouse’s head, and the focal plane was adjusted manually to show a clear image of the vessels. One piece of self-gripping fastener material was taped to the battery, and the complementary piece was sutured to the back of the mouse. Subsequently, the wScope body and battery were connected with a soft thin wire. The gain and LED power were adjusted to the proper values (vessel is clearly visible, and no over exposure), and the device was powered down immediately to conserve battery life. All these adjustments were made by remote control. Then, the anesthetized animal was transferred to the experimental arena. The wScope was wirelessly turned on 10 min after waking. The behavior of the mouse was observed by the experimenters, and visual stimuli were presented for 2 s when the animal’s head was turned toward one of the screens placed behind two transparent walls of the arena.

After the presentation of stimuli, the vessels in V1 quickly began to dilate (0.45  s±0.11) and reached their peak dilation amplitude (19.46%±4.21) in 1.66  s±0.10, followed by contraction in 5.69  s±0.36 to reach their peak contraction amplitude (7.70%±1.57), after which they gradually returned to baseline [Figs. 3(b)–3(d)]. The vascular kinetics were correlated with visual stimulation, and the amplitude of the vessel dilations was significantly higher than that of the contractions. Our data are well consistent with reported works.^18^^–^^20^ These results demonstrate that our wireless microscope system can reliably record the changes in cerebral vessels in freely moving mice, enabling brain hemodynamic imaging studies for a variety of topics, such as cerebrovascular disorders and neurovascular kinetics.

The wScope has similar imaging performance to existing wired microscopes, while its wireless hardware allows the experimenter to study the behavior of multiple freely moving mice at once; it can even be used while the animals are in enclosed space (Fig. S3 in the Supplementary Material). Using wScopes, we successfully imaged 4 mice simultaneously in the same arena (Figs. S4 and S5 in the Supplementary Material). We further compared these data with that from previously published literature on cerebral hemodynamic changes in response to stimuli; our data were comparable to those in the literature, demonstrating the reliability of our system.

### 3.4 *In Vivo* Recording the neuronal Activation Study Simultaneously

**Fig. 4:**
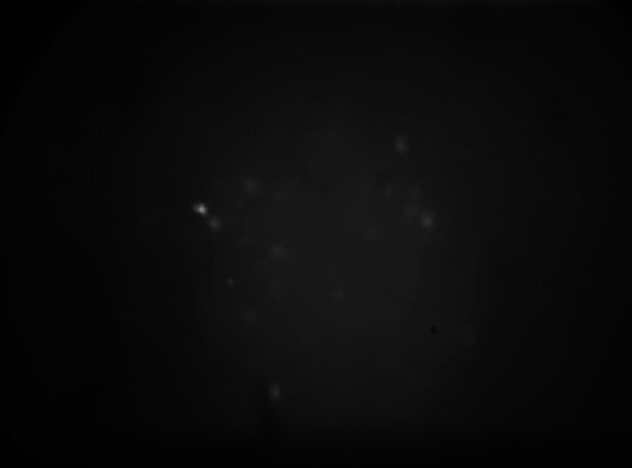
Example of GCaMP fluorescence signals in mouse (Video 1, MP4, 7.91 MB [URL: https://doi.org/10.1117/1.JBO.28.2.026503.s1]).

To demonstrate the *in vivo* real-time imaging performance for recording the neuron activities, we first imaged neurons in mice cortex. A virus (AAV2/9.syn.GCaMP6m.WPRE.SV40, diluted to a titer of $4\times 10^{12}\;\mathrm{vg}/\mathrm{ml}$) was drawn into a prepared glass pipette (tip size: 0.3 to 0.5 mm) through negative pressure. To create a seal against the surface of the brain, the glass pipette was slowly lowered again by ∼450  μm to press on the pia without breaking through it. Viral infusion was started after 2 min and proceeded at 60  nl/min for 10 min.^21^ The craniotomy was covered with a small piece of glass coverslip after infusion. The animals recovered for 7 days after the craniotomy and were intraperitoneally administered ceftriaxone sodium (200  mg/kg) every day to prevent inflammation. These results show that our system has good sensitivity and resolution to study GCaMP fluorescence signals in mouse (Fig. 4).

**Fig. 5:**
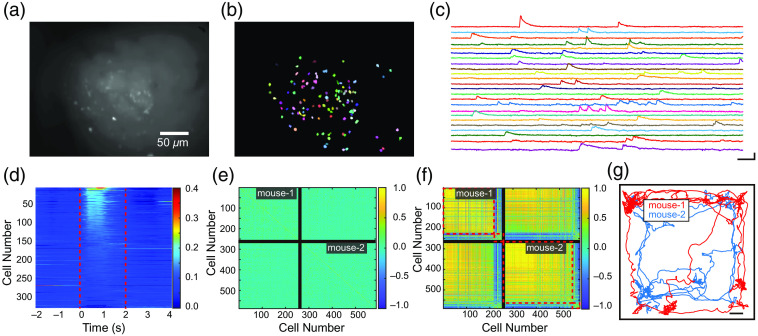
Primary visual cortex imaging in two freely moving mice. (a) Average projection image from a 5-min recording session. (b) Extracted contours of neurons using CNMF-E, colored randomly. (c) Representative transient calcium traces extracted from the time-series data. (d) Heatmap of neuronal calcium transients during visual stimulation. The red dashed lines indicate the stimulus phase. (e) Matrix of the Pearson correlation coefficients between the calcium transients of pairs of neurons from the two mice during the dark phase. (f) Matrix of the Pearson correlation coefficients between the calcium transients of different neurons from the two mice during the dark phase (left) and the stimulus phase (right). The black line separates the neurons of two mice from each other; the red dashed lines group highly correlated neural populations. (g) Locomotion trajectories of two mice fitted with the wireless microscope system within the arena (right, Scale bar: 5 cm).

We next presented the performance of the system in simultaneously recording neuronal activity from another two free-moving mice. The location of V1 was determined using stereotactic coordinates. Four weeks after the injection of rAAV2/9-hsyn-GCamp6m in V1, the mice were trained using dummy microscopes for one week. During the experiments, the mice were allowed to freely explore and engage in social interaction in the arena. The arena and visual stimuli were the same as those in Fig. 3(a). The population neuronal activity in V1 was analyzed using extended constrained nonnegative matrix factorization (CNMF-E) and ImageCN.^21^^–^^23^ The spatial components were extracted from the time-series data [Fig. 5(a) and 5(b)]. The temporal components of neuronal activities were also extracted, and calcium signals were distinguishable from the background noise [Fig. 5(c)]. The neural population in the V1 areas of mice demonstrated strong activity beginning ∼0.1  s after the onset of stimulus presentation [Fig. 5(d)]. To evaluate the performance of the wireless system in capturing the two recordings independently and simultaneously and confirm the absence of interference between them, we used the absolute value of the Pearson correlation coefficient, a statistic for measuring the linear relationship between each pair of neurons, to quantify the inter- and intragroup correlations of the neuron populations. The population correlation between the two mice was low during the dark phase, the signal received by two mice were independent, indicating that no interference was detected during simultaneous recording with two microscopes [Fig. 5(e)]. However, during visual stimulus presentation, the neuron populations tended to fire simultaneously. Thus, the correlation of activity between pairs of neurons in both mice was higher in this phase than during the dark phase, when these populations were quiescent [Fig. 5(f)]. The behavior of the animals was recorded with a camera; the corresponding movement trajectories of the two mice are shown in Fig. 5(g). Overall, we recorded the calcium transients of V1 neurons in two mice at the same area simultaneously, and no signal interference was observed between different channels of microscopes, indicating that our system provides a powerful tool for recording the activity of neural populations in multiple freely moving mice with excellent spatial and temporal resolution.

## 4 Discussion

In the current version of the wScope, wireless transmission was realized using analog signal FM. This analog scheme has relatively low resistance to environmental interference and has low sensitivity to weak signal changes, so the system needs to be used in a relatively clean electromagnetic environment. On the other hand, although digital transmission schemes that are currently in common use, such as Wi-Fi and Bluetooth, it is still very challenging to use on shelf parts in wireless miniscope. A major barrier is that those on shelf digital wireless components, especially related chips, generally have a much higher power consumption, as well as larger size and heavier weight. With the advancement of semiconductor technology and chip design, digital wireless transmission solutions with ultralow power consumption are possible in future.

For the power source, the development of new battery technology will also extend the working life of the system. Another potential breakthrough solution is the future advancement of wireless charging technology, which can make it possible to eliminate battery-life limitations completely and monitor neuronal activity in the long term, e.g., days or months. These capabilities will create exciting new opportunities.

Miniaturized microscopes play an important role in neuroscience, helping researchers study neural circuits in freely behaving animals. Our wireless microscope system could extend the range of applications for brain imaging in freely moving rodents, including some animal experiments are conducted in covered area, such as caves, which would not permit the use of wired microscopes. Furthermore, the wScope can be used in free-moving larger animals, such as primates, that cannot use the tethered cable. Even more, with a larger brain size and body weight, multiple wScopes could be mounted on one head that allows the study of cross-brain neuron connections in free-moving large primates.

## 5 Conclusion

In this paper, we developed a wireless miniaturized microscope system for simultaneous brain imaging in multiple mice. Using FM technology, wireless signal transmission and system control were achieved.

Our system has a smaller mass (2.7 g), higher resolution (640×480  pixels) and a faster frame rate (25 Hz) than other recently reported wireless microscope systems. Compared with on board SD card solution, our system can provide important real-time previewing function. Moreover, we added the ability to remotely control imaging parameters and switch the device between standby mode and recording mode to improve the imaging performance of our system and extend its battery life. As shown by our data, this wireless system achieved comparable imaging quality with wired mini-scopes, and chronologically recorded images of CBF and neuronal activity in V1 with high fidelity and resolution. We believe that with the advances in chip and battery technology, wireless mini-scopes will play more important roles for neuroscience studies.

## References

[1] Ghosh K. K. et al., “Miniaturized integration of a fluorescence microscope,” Nat. Methods 8(10), 871–878 (2011).1548-7091 10.1038/nmeth.1694 21909102 PMC3810311

[2] Cai D. J. et al., “A shared neural ensemble links distinct contextual memories encoded close in time,” Nature 534(7605), 115–118 (2016). 10.1038/nature17955 27251287 PMC5063500

[3] Skocek O. et al., “High-speed volumetric imaging of neuronal activity in freely moving rodents,” Nat. Methods 15(6), 429–432 (2018).1548-7091 10.1038/s41592-018-0008-0 29736000 PMC7990085

[4] Rynes M. L. et al., “Miniaturized head-mounted microscope for whole-cortex mesoscale imaging in freely behaving mice,” Nat. Methods 18(4), 417–425 (2021).1548-7091 10.1038/s41592-021-01104-8 33820987 PMC8034419

[5] Liberti W. A. et al., “An open source, wireless capable miniature microscope system,” J. Neural Eng. 14(4), (2017).1741-2560 10.1088/1741-2552/aa6806 PMC5955387 28514229

[r6] Kingsbury L. et al., “Correlated neural activity and encoding of behavior across brains of socially interacting animals,” Cell 178(2), 429–446.e16 (2019). CELLB5 0092-8674 10.1016/j.cell.2019.05.022 31230711 PMC6625832

[r7] Karigo T. et al., “Distinct hypothalamic control of same- and opposite-sex mounting behaviour in mice,” Nature 589(7842), E9–E9 (2021). 10.1038/s41586-020-03143-1 33408419

[r8] Kennedy A. et al., “Stimulus-specific hypothalamic encoding of a persistent defensive state,” Nature 586(7831), 730 (2020). 10.1038/s41586-020-2728-4 32939094 PMC7606611

[9] Shin J. H. et al., “Spatial organization of functional clusters representing reward and movement information in the striatal direct and indirect pathways,” Proc. Natl. Acad. Sci. U. S. A. 117(43), 27004–27015 (2020). 10.1073/pnas.2010361117 33055217 PMC7604453

[10] Shuman T. et al., “Breakdown of spatial coding and interneuron synchronization in epileptic mice,” Nat. Neurosci. 23(2), 229–238 (2020). NANEFN 1097-6256 10.1038/s41593-019-0559-0 31907437 PMC7259114

[11] Barbera G. et al., “A wireless miniScope for deep brain imaging in freely moving mice,” J. Neurosci. Methods 323, 56–60 (2019). JNMEDT 0165-0270 10.1016/j.jneumeth.2019.05.008 31116963 PMC6636826

[12] Thomos N. Boulgouris N. V. Strintzis M. G., “Optimized transmission of JPEG2000 streams over wireless channels,” IEEE Trans. Image Process. 15(1), 54–67 (2006). IIPRE4 1057-7149 10.1109/TIP.2005.860338 16435536

[13] Setiadi D. I. M., “PSNR vs SSIM: imperceptibility quality assessment for image steganography,” Multimedia Tools Appl. 80(6), 8423–8444 (2021). 10.1007/s11042-020-10035-z

[14] Zhou W. et al., “Image quality assessment: from error visibility to structural similarity,” IEEE Trans. Image Process. 13(4), 600–612 (2004). IIPRE4 1057-7149 10.1109/TIP.2003.819861 15376593

[15] Horé A. Ziou D., “Image Quality Metrics: PSNR vs. SSIM,” in 20th Int. Conf. Pattern Recognit., pp. 2366–2369 (2010).

[16] Li X. Cai J., “Robust transmission of JPEG2000 encoded images over packet loss channels,” in IEEE Int. Conf. Multimedia and Expo, pp. 947–950 (2007). 10.1109/ICME.2007.4284808

[17] Tian Y. L. et al., “An excitatory neural assembly encodes short-term memory in the prefrontal cortex,” Cell Rep. 22(7), 1734–1744 (2018). 10.1016/j.celrep.2018.01.050 29444427

[18] Uhlirova H. et al., “Cell type specificity of neurovascular coupling in cerebral cortex,” Elife 5, e14315 (2016). 10.7554/eLife.14315 27244241 PMC4933561

[r19] Kisler K. et al., “*In vivo* imaging and analysis of cerebrovascular hemodynamic responses and tissue oxygenation in the mouse brain,” Nat. Protoc. 13(6), 1377–1402 (2018).1754-2189 10.1038/nprot.2018.034 29844521 PMC6402338

[20] O’Herron P. et al., “Neural correlates of single-vessel haemodynamic responses in vivo,” Nature 534(7607), 378 (2016). 10.1038/nature17965 27281215 PMC4911280

[21] Wang Y. Z. et al., “Efficient implementation of convolutional neural networks in the data processing of two-photon *in vivo* imaging,” Bioinformatics 35(17), 3208–3210 (2019). BOINFP 1367-4803 10.1093/bioinformatics/btz055 30689714 PMC6735786

[r22] Zhou P. et al., “Efficient and accurate extraction of *in vivo* calcium signals from microendoscopic video data,” eLife 7, e28728 (2018). 10.7554/eLife.28728 29469809 PMC5871355

[23] Pnevmatikakis E. A. et al., “Simultaneous denoising, deconvolution, and demixing of calcium imaging data,” Neuron 89(2), 285–299 (2016). NERNET 0896-6273 10.1016/j.neuron.2015.11.037 26774160 PMC4881387

