# Supplementary material for: Cable-free brain imaging for multiple free-moving animals with miniature wireless microscopes
Source: J Biomed Opt. 2023 Feb 10;28(2):026503. doi: 10.1117/1.JBO.28.2.026503 (PMC9917720; doi:10.1117/1.JBO.28.2.026503)
Supplement: Supplementary file 1 [file JBO_028_026503_SD001.pdf]

Table S1. Battery Information

Size	Capacity	Weight
4.0mm*10mm*15mm	60mAh	1.32g
4.0mm*10mm*25mm	100mAh	1.7g
6.0mm*12mm*25mm	130mAh	3.3g

Figure S1. Wireless microscope control program user interface

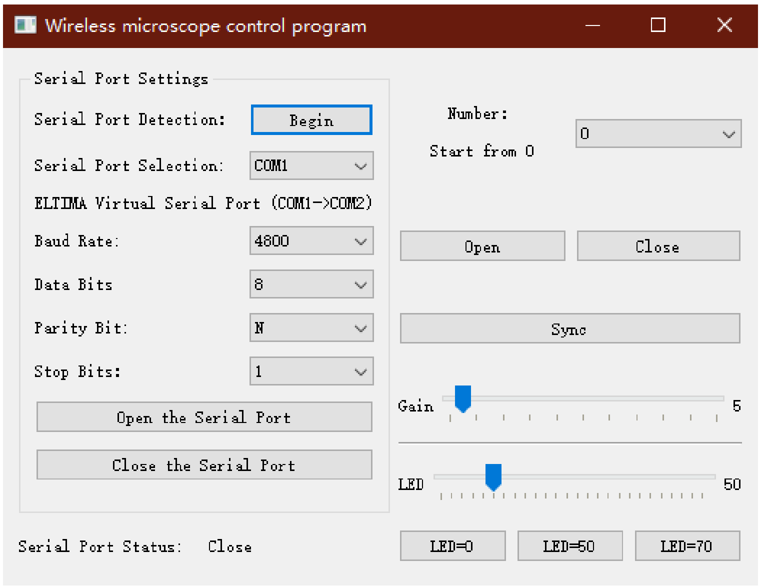

Figure S2. Animal recovery training using dummy microscope after surgery

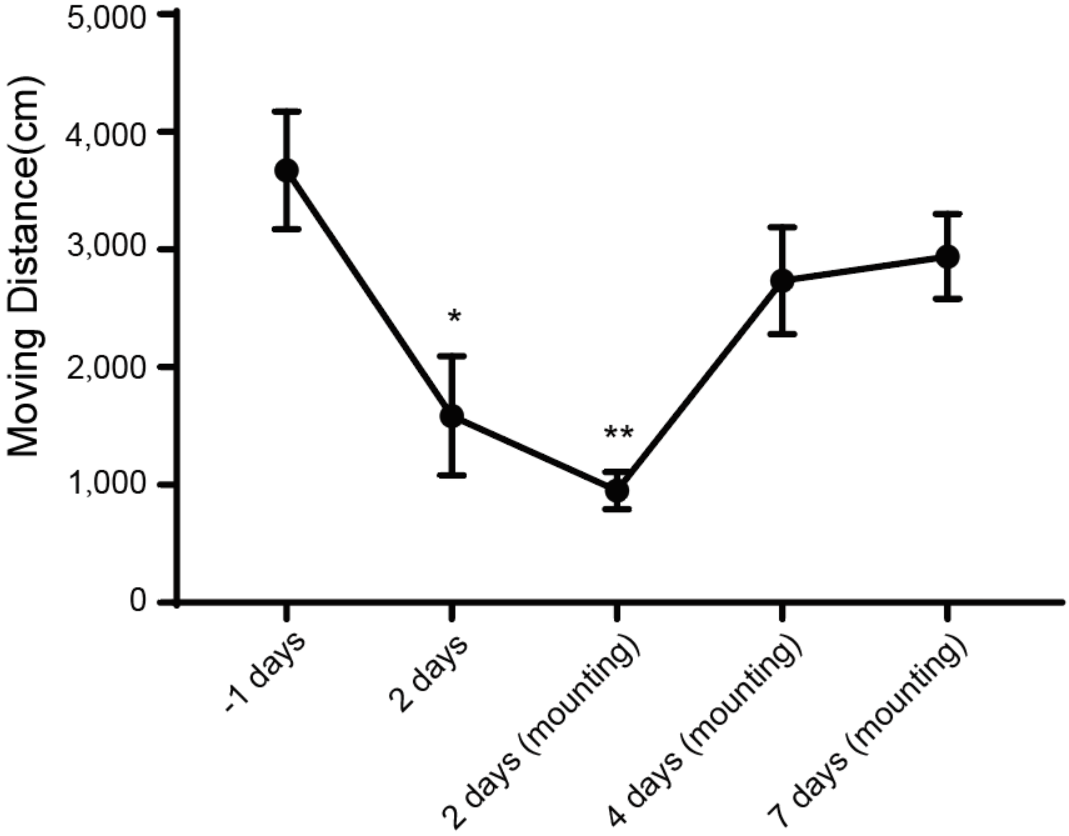

Animal moving distance in arena for 10 minutes was calculated to evaluate the effects of the surgery and the mounting of microscope to mice. 1 day before surgery, the moving distances were calculated as control. 2 days after surgery, the moving distances were decreased significantly, and a further decreased was observed after the mounting of dummy microscope. After 4 days training, the behavior of mice was recovered.

Figure S3. Evaluation imaging PSNR while the animal was in enclosed space

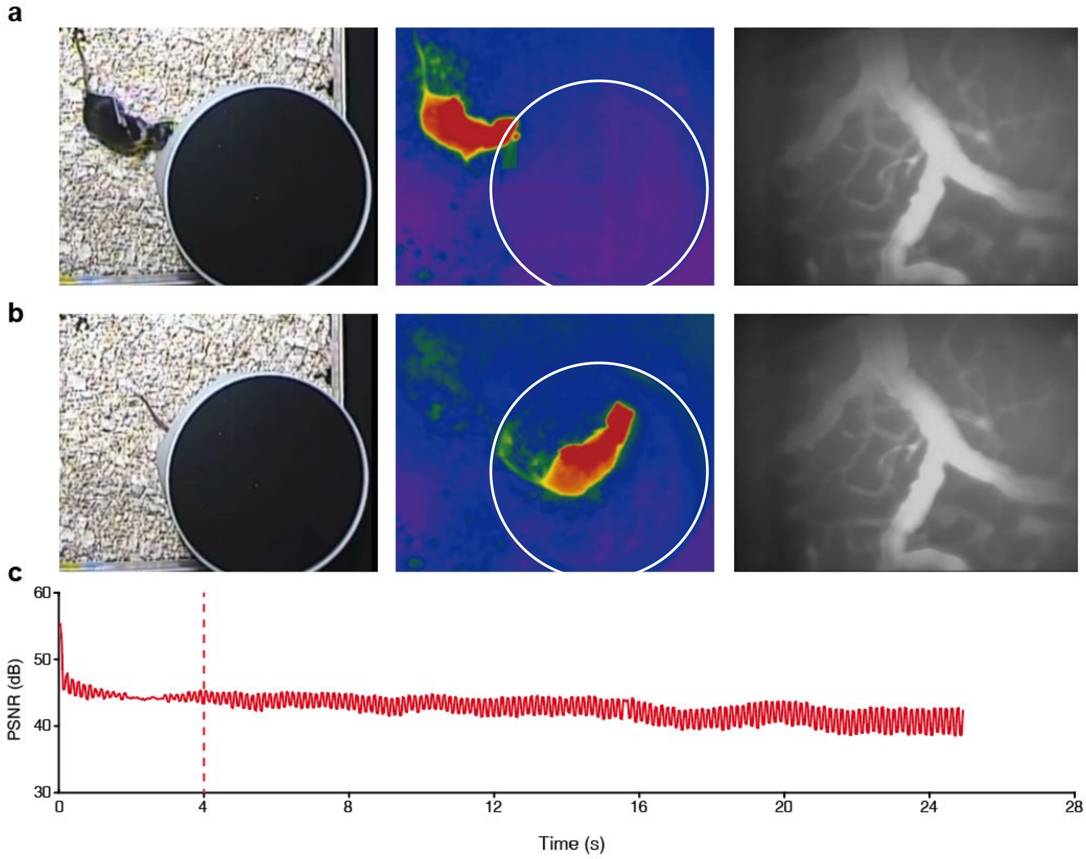

a. Mouse mounted with wScope was exploring the door of the enclosed space. Left: visible light behavior camera imaging, middle: thermal infrared behavior camera imaging, right: vessel imaging using wScope; **b**. Same depiction as a, except the body of mouse was in inside the enclosed space; **c**. PSNR of vessel images before and after the mouse entered the enclosed space, as compared to the first frame, red dash line denotes the moment of mouse's head is entering the space.

Figure S4. Simultaneously 4 mice locomotion recording in the same arena

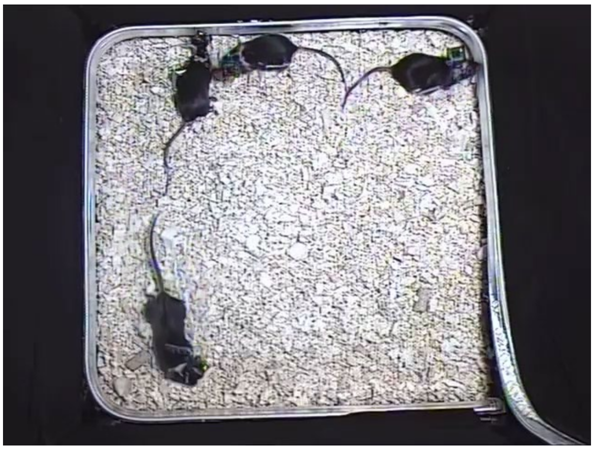

4 mice mounted with wScopes were freely locomoting in the same arena simultaneously.

Figure S5. Cerebral vessel imaging in 4 mice simultaneously

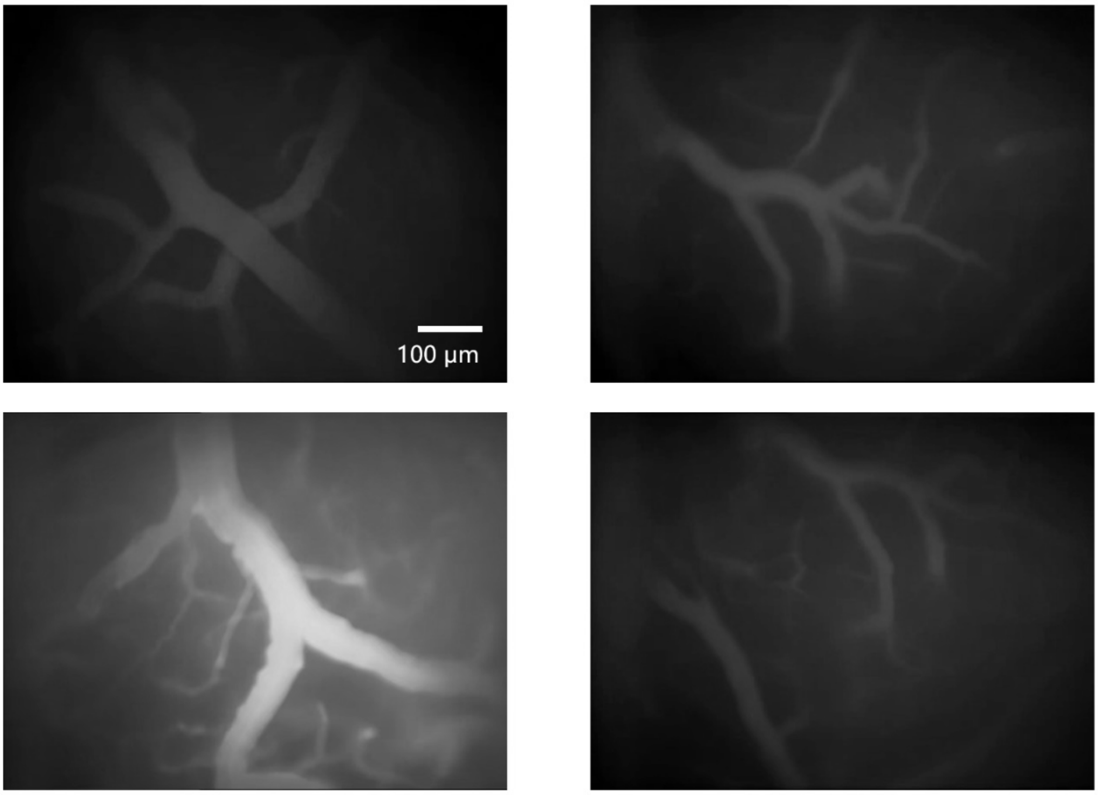

4 mice brain vessels imaging simultaneously using wScope.
